# Editorial: Domestication and evolution in dogs: current issues and questions that remain

**DOI:** 10.3389/fpsyg.2024.1455973

**Published:** 2024-07-16

**Authors:** Katherine E. Bruce, David A. Leavens, Sarah T. Boysen

**Affiliations:** ^1^Department of Psychology, University of North Carolina at Wilmington, Wilmington, NC, United States; ^2^School of Psychology, University of Sussex, Brighton, United Kingdom; ^3^Comparative Cognition Project, Sunbury, OH, United States

**Keywords:** domestication, evolution, dogs, canids, behavior

There is considerable research interest related to domestication and evolution of dogs and areas of concentration center around a variety of larger topics. The Research Topic, entitled “*Domestication and Evolution in Dogs: Current Issues and Questions That Remain*,” includes four articles highlighting different issues currently being studied. Each of the articles features analyses related to the human-canid bond. Two of the articles include comparisons to canids other than domesticated dogs- wolves and dingoes; one features analyses of human personality and interactions with shelter dogs; and the last showcases a non-invasive procedure to investigate the relation between DNA methylation and genotypes and dog behavior. Each article summarized below serves as a focal point to spark additional research in these areas.

In the initial article, Burkhard et al. used a survey approach to investigate how different experiences of human trainers with dogs or wolves housed at the Wolf Science Center in Austria differed in terms of predicting perceived bonds with the canids. Further, once these results were obtained, canids were observed in a behavioral “greeting” test to see whether they reacted to the trainers in manner consistent with the trainer's perception. Each trainer completed a survey assessing their perceived bond with the wolves and dogs at the park; in addition, trainers rated their perceptions of the bonds that other trainers had with the animals. Later, social and agonistic behaviors were scored when the canids were allowed to interact with the trainer in a short greeting test. Burkhard et al. noted that only the trainer's experience of hand-raising a specific animal was significantly associated with perceptions of a strong human-canid bond with that animal, regardless of whether the animal was a wolf or a dog. Further, this perception of a strong bond predicted the animal's staying in proximity to the trainer in the greeting test. Using factor analysis, other characteristics, such as sex of the animal (males more than females stayed in proximity to a preferred trainer) and years of trainer experience, predicted affiliative behavior. Canid species was not a significant factor. While the sample size was small, these data supported the Canine Cooperation Hypothesis that, with socialization, wolves can accept humans as social partners similar to dogs rather than the Hypersociabilty Hypothesis that predicts species differences in the human-canid bond with dogs more social than wolves.

The second article by Brumm et al. makes an interesting case for a likely process of domestication of wolves. The authors begin by comparing the two prevailing hypotheses about wolf domestication to produce dogs, that is whether the process was wolf-initiated or human-initiated, and in the remainder of the article argue the likelihood of a human-initiated process. They employ two lines of evidence- first, comparison to aboriginal dingo associations and second, archaeological evidence of the Late Pleistocene.

Also considering the relationship between human personality and dog behavior, Shih et al. described the correlations between personality characteristics of animal shelter volunteers and their interactions walking shelter dogs. Researchers measured personality using the NEO Five Factor Personality Inventory and noted consistent associations between neuroticism, extroversion, openness, agreeableness and conscientiousness and how the dogs behaved on-leash and how the volunteers treated the dogs (vocalizations, praise, tightness of leash, etc.). Shih et al. suggest that these findings could be used to pair volunteers and shelter dogs for more effective and positive interactions.

Finally, Sanders et al. present an analysis of associations between dog behaviors reported by their owners (measured by the C-BARQ) and specific DNA methylation and genotypes (collection via buccal swabs). After controlling for age of the 46 dogs, they found that their energy and stranger-directed fear had significant associations with DNA methylation. This behavioral epigenetic research approach should spark additional important research in this area.

## Author contributions

KB: Writing – original draft. DL: Writing – review & editing. SB: Writing – review & editing.

